# Control of the Electroporation Efficiency of Nanosecond Pulses by Swinging the Electric Field Vector Direction

**DOI:** 10.3390/ijms241310921

**Published:** 2023-06-30

**Authors:** Vitalii Kim, Iurii Semenov, Allen S. Kiester, Mark A. Keppler, Bennett L. Ibey, Joel N. Bixler, Ruben M. L. Colunga Biancatelli, Andrei G. Pakhomov

**Affiliations:** 1 Frank Reidy Research Center for Bioelectrics, Old Dominion University, Norfolk, VA 23508, USA; 2 Bioeffects Division, Airman System Directorate, 711th Human Performance Wing, Air Force Research Laboratory, JBSA Fort Sam Houston, San Antonio, TX 78234, USA; 3 SAIC, San Antonio, TX 78234, USA; 4 Department of Physiological Sciences, Eastern Virginia Medical School, Norfolk, VA 23508, USA

**Keywords:** electroporation, electropermeabilization, nanosecond pulses, nsEP, focal electroporation, cell membrane polarization

## Abstract

Reversing the pulse polarity, i.e., changing the electric field direction by 180°, inhibits electroporation and electrostimulation by nanosecond electric pulses (nsEPs). This feature, known as “bipolar cancellation,” enables selective remote targeting with nsEPs and reduces the neuromuscular side effects of ablation therapies. We analyzed the biophysical mechanisms and measured how cancellation weakens and is replaced by facilitation when nsEPs are applied from different directions at angles from 0 to 180°. Monolayers of endothelial cells were electroporated by a train of five pulses (600 ns) or five paired pulses (600 + 600 ns) applied at 1 Hz or 833 kHz. Reversing the electric field in the pairs (180° direction change) caused 2-fold (1 Hz) or 20-fold (833 kHz) weaker electroporation than the train of single nsEPs. Reducing the angle between pulse directions in the pairs weakened cancellation and replaced it with facilitation at angles <160° (1 Hz) and <130° (833 kHz). Facilitation plateaued at about three-fold stronger electroporation compared to single pulses at 90–100° angle for both nsEP frequencies. The profound dependence of the efficiency on the angle enables novel protocols for highly selective focal electroporation at one electrode in a three-electrode array while avoiding effects at the other electrodes. Nanosecond-resolution imaging of cell membrane potential was used to link the selectivity to charging kinetics by co- and counter-directional nsEPs.

## 1. Introduction

Electroporation, also known as electropermeabilization, is a common technique to compromise cell membrane integrity, with applications in fundamental membrane biophysics studies, gene and drug delivery, tissue and tumor ablation, and in the food industry [1,2,3,4,5,6,7,8,9]. Applying the external electric field polarizes the cell, with the highest transmembrane potential induced at the cell poles facing the electrodes, while membrane areas orthogonal to the electric field direction (“equator”) remain unaffected. Hence, applying electric pulses (EPs) from different directions can permeabilize a larger cell surface, thereby increasing the efficiency of electroporation treatments. Indeed, changing the electric field vector direction by 90° enhanced electroporation-mediated dye entry [10], tumor ablation by electrochemotherapy [11], and gene electrotransfer in vitro [10], although findings in vivo were less definitive [12]. “Rotating” the electric field by energizing sequentially different pairs of electrodes in a circle array had higher therapeutic efficiency for murine B16 melanoma compared to several other electrode configurations [13]. Applying 5 ms pulses from opposite directions (180° vector change) increased the transfected cell number by 40% compared to no direction change [10]. The sequence of how multiple EPs are delivered by multi-electrode arrays was optimized to electroporate cells from different directions and maximize the treatment efficiency [14,15]. In other applications, cells were electroporated by a long pulse in a flow chamber where cells are rotated while floating along the electrodes to expose different portions of the cell membrane to the electric field [16].

The effect of direction change becomes more complex when EP duration is decreased into the nanosecond and short microsecond range. Applying a second nanosecond EP (nsEP) in the direction opposite to a preceding first nsEP inhibits (or “cancels”) the anticipated effects of the first nsEP [17,18,19,20,21,22,23]. As a result, the electroporation and electrostimulation efficiency of a pair of opposite-direction pulses is smaller than of the first nsEP alone, despite delivering more energy. The biophysical mechanisms of this “bipolar cancellation” effect are not fully understood despite extensive research. It has recently become clear that the bipolar cancellations of neurostimulation and electroporation have different dependences on pulse parameters and are likely caused by different mechanisms; there may also be a difference in cancellation mechanisms for electroporation by the shortest nsEPs (a few nanoseconds) and EPs in the range of hundreds of nanoseconds to microseconds [17].

All published studies of bipolar cancellation have been limited to a 180° change in the electric field direction, by applying either bipolar pulses or two separate pulses in the opposite direction. The presence and efficiency of cancellation at smaller angles has not been tested. This knowledge is critically important for the selective distant targeting of nsEP effects (such as in “CANCAN” protocols [24,25]) and for understanding the underlying biophysical processes. Intuitively, when the angle nears 0°, the scenario is equivalent to simply doubling nsEP duration, which would obviously facilitate electroporation and electrostimulation effects. Here, we explored how the cancellation of electroporation depends on the angle by which the electric field direction is changed in a pair of nsEPs, and at what angle it is replaced by facilitation. We applied several pulse pairs so that the electric field vector was alternating (“swinging”) between two directions. The measurements were performed both at 1 Hz and at a high repetition rate of 833 kHz, which markedly enhanced cancellation for the 600 + 600 ns pulse pairs [17]. The experiments revealed a remarkably strong dependence of electroporation efficiency on the angle, especially for the high-rate pulse trains. Counter-directional nsEPs caused alternating brief polarizations and depolarizations of the cell membrane, while co-directional nsEPs coalesced into a long, higher-amplitude polarization with expectedly much stronger effects. This strong dependence of electroporation on the angle enables a novel paradigm of achieving focal effects at one electrode while avoiding them at other electrodes in triangle and pyramid-like electrode configurations. This novel method is promising for avoiding the adverse side effects of neuromuscular stimulation in localized tumor ablation and gene electrotransfer treatments.

## 2. Results

### 2.1. Applying nsEPs from Different Directions Can Inhibit or Facilitate Electroporation

**Figure 1:**
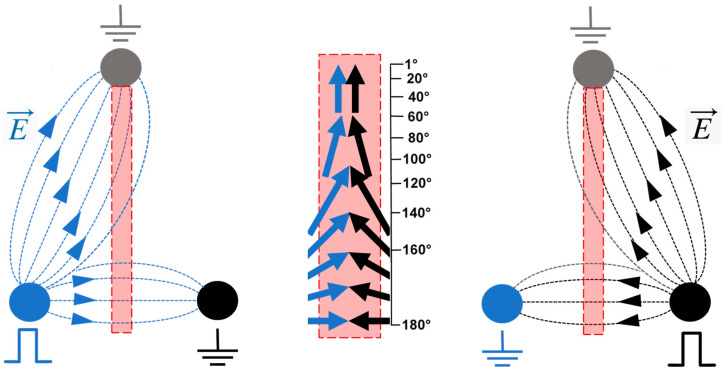
A schematic explaining how a triangular electrode array was employed to change the electric field vector direction. (**Left panel**) shows the electric field lines when one base electrode is energized and two other electrodes are grounded. (**Right panel**) shows a symmetrical situation with the right base electrode energized. When the base electrodes are energized in alternation, the electric field vector direction changes. Along the bisecting line (filled rectangle, expanded in the (**center panel**)), the angle of the vector direction change gradually decreases from 180° at the base to nearly 0° near the apex.

A quantitative analysis of the impact of the angle at which the electric field direction is changed was performed in BPAE cell monolayers by measuring the uptake of a membrane permeability marker dye YO-PRO-1 (YP). Three electrodes made of hollow needles and forming a triangle were placed orthogonal to the monolayer. When one base electrode was energized, the electric field lines were directed towards the apex electrode and the second base electrode (Figure 1, left panel). Energizing the second base electrode and making the first one ground mirrored the electric field lines (Figure 1, right panel). When a pair of pulses was applied to energize one base electrode right after the other, the cells experienced a location-dependent change in the electric field direction. Specifically, along the bisecting line (Figure 1, center), the electric field vector angle gradually changed from 180° at the base to nearly 0° at the apex electrode. This setup enabled testing the entire range of different angles in a single experiment by selecting multiple regions of interest (ROIs) along the bisecting line. In the control experiments, only one electrode was energized, and cells in each ROI were exposed to a single pulse of the same intensity (5 to 10 kV/cm) as with paired pulses.

**Figure 2:**
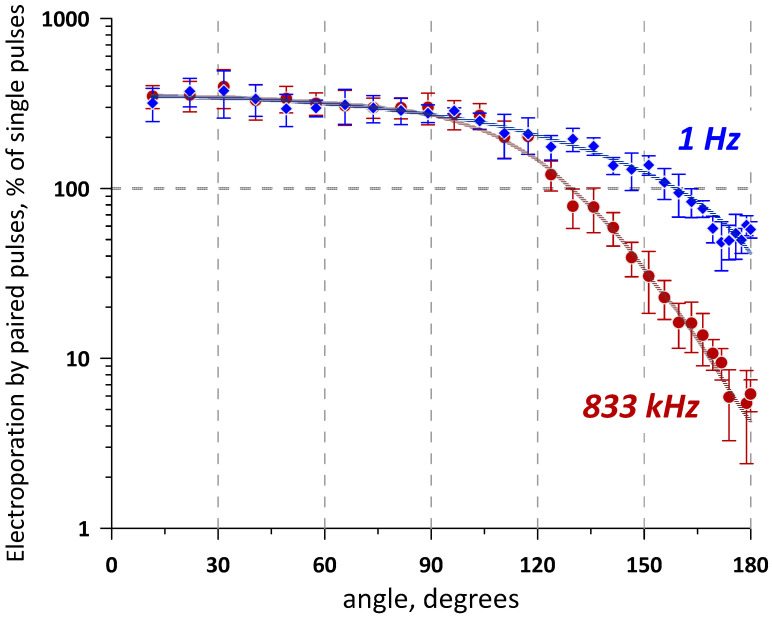
Effect of the electric field vector change on the electroporation efficiency of paired-pulse trains (5 pulses, 600 + 600 ns) compared to single-pulse trains (5 pulses, 600 ns). Pulses or pulse pairs were applied to a BPAE cell monolayer at either 1 Hz or 833 kHz (legends). Abscissa is the angle at which the electric field vector direction changed between two pulses in a pair. Ordinate is the resulting change in the electroporation efficiency, measured as change in YP emission compared to the effect of a single-pulse train in the same region of interest (taken as 100%). Note that applying pulses in pairs enhanced electroporation at small angles but inhibited them at large angles in a frequency-dependent manner. Mean ± s.e., n = 3.

Trains of five pulses (600 ns) or five paired pulses (600 + 600 ns) were delivered either at a low 1 Hz frequency or at the maximum frequency of 833 kHz (no delay between the pairs). YP uptake was averaged within 32 ROI placed along the bisection line. The values measured in control samples at 1 Hz and 833 kHz were taken as 100% for their respective treatments with paired pulses. The experiments show a strong dependence of YP uptake on the angle of the electric field direction change (Figure 2). Maximum cancellation was expectedly observed at the 180° vector change, reducing YP uptake compared to single-pulse trains 2-fold at 1 Hz and as much as 20-fold at 833 kHz. Stronger cancellation at sub-MHz frequencies was consistent with our previous report using bipolar pulses [17]. Cancellation weakened as the angle was reduced, and at about 160° (1 Hz) or 130° (833 kHz), the effects of paired-pulse trains became the same as those of single-pulse ones. Further reduction of the angle enhanced electroporation, up to about three-fold at 90–100° for both nsEP frequencies, followed by a plateau all the way to 0°.

At angles close to 0°, co-directional nsEP pairs are “seen” by cells as pulses of double duration, which are expectedly more efficient. However, at a 90° angle, they should act as two separate 600 ns pulses that electroporate different regions of the cell membrane. Applying conventional (“long”) pulses with a 90° direction change typically enhanced electroporation effects [10,11], and observing a plateau from 0 to 90° was unexpected. This finding was validated in a separate series of experiments by directly comparing the effects of two co-directional pulses and two orthogonally directed pulses.

### 2.2. No Change in Electroporation When the Electric Field Direction Is Rotated by 90°

These experiments utilized a square electrode array, where paired pulses (600 + 600 ns with a 50 ns interval) were applied either along a single diagonal of the array or alternated between two diagonals (see Section 4.2 and Section 4.3 for more details). A train of four paired pulses at either 1 Hz or 770 kHz delivered the electric field of 10 kV/cm to the center of the array. YP emission was measured only in the center of the array, where pulses in each pair were either co-directional or cross-directional (i.e., came at 90° angle).

**Figure 3:**
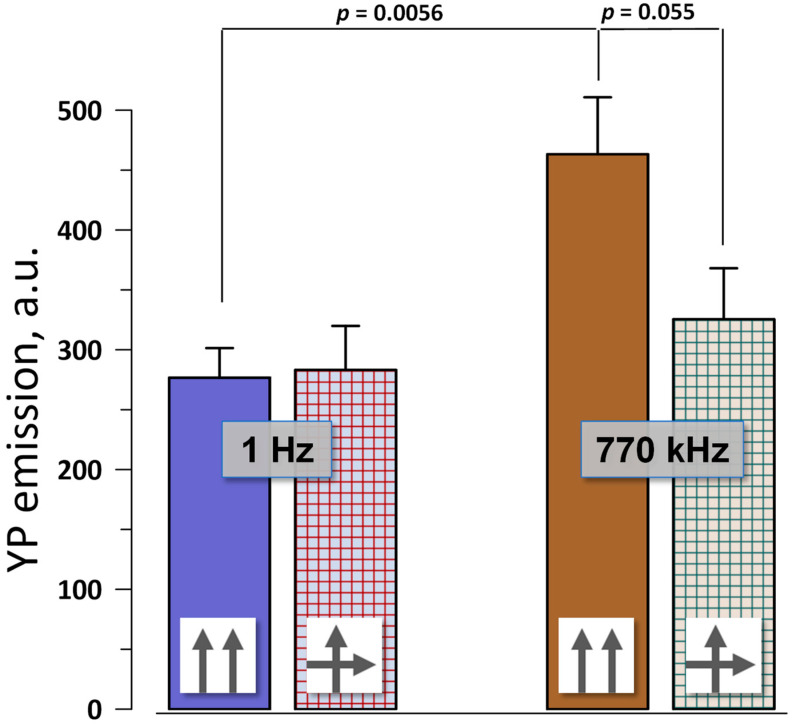
Electroporation efficiency of co-directional and cross-directional paired pulses. Trains of 4 paired pulses (600 + 600 ns) were applied to BPAE cell monolayers at either 1 Hz or 770 kHz, at 10 kV/cm. The electric field direction either stayed the same in each pair (↑↑) or was changed by 90° (↑→). Electroporation efficiency was measured by the uptake of YP dye. Mean ± s.e., n = 6. *p*-values were calculated with two-tailed Student’s *t*-test. See text for more details.

Consistently with the measurements in Figure 2, co- and cross-directional pulses at 1 Hz had the same electroporation efficiency (Figure 3). Increasing the pulse frequency to 770 kHz enhanced electroporation by the co-directional pulses because of the temporal summation of the induced membrane potential [17,26,27,28]. Interestingly, charging two different regions of the cell membrane did not cause summation, making cross-directional pulses at 770 kHz just as effective as at 1 Hz. Contrary to data with “long” pulses [10,11], electroporative dye uptake after nsEP did not depend on whether we electroporated the same or a larger area of the cell membrane.

### 2.3. Focal Electroporation in the Triangular Array

**Figure 4:**
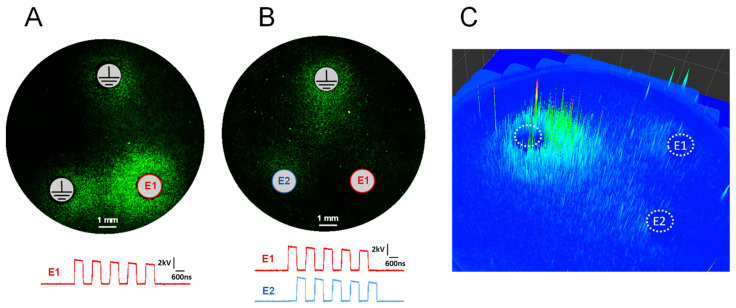
The difference in electroporation patterns produced by a train of single pulses ((**A**), 5 × 600 ns) and a train of paired pulses ((**B**,**C**), 5 × 600 + 600 ns) at 833 kHz. BPAE cell monolayers were electroporated using a triangular electrode array in the presence of YP dye. All panels are fluorescence images of YP emission (green), with electrode positions labeled. Panel (**C**) is a 3D representation of YP uptake pattern. Insets in (**A**,**B**) show the oscilloscope traces of pulses applied to either E1 electrode only (**A**) or in alternation to E1 and E2 electrodes (**B**).

The profound, 60-fold difference in electroporation efficiency of co- and counter-directional nsEPs at 833 kHz (Figure 2) produced a unique “unipolar” pattern of electroporation. In control cell monolayers exposed to a train of five single pulses applied to one of the base electrodes (833 kHz, 4 kV), the distribution of YP fluorescence (Figure 4A) simply followed the electric field strength map (see Section 4.3). In contrast, energizing two base electrodes in alternation (five paired pulses, 600 + 600 ns, 833 kHz, 4 kV) produced a strong effect near the apex electrode (ground) but weak or no effect near the base electrodes, despite the strong electric field there (Figure 4B,C). Indeed, co-directional nsEPs near the apex “coalesced” into a long pulse, which was efficient at electroporation because of its long duration, single polarity, and sufficiently high electric field strength. In contrast, counter-directional nsEPs near the base electrodes had low efficiency despite the high electric field strength, because of their nanosecond duration and bipolar pulse shape. These explanations were validated by measuring the kinetics of cell membrane charging by nsEP trains at different locations within the triangular array.

### 2.4. Cell Membrane Charging by Co- and Counter-Directional nsEPs

We employed strobe photography with FluoVolt voltage-sensitive fluorescent dye for nanosecond-resolution monitoring of cell membrane charging by nsEP trains. In FluoVolt-loaded cells exposed to electric field pulses, emission is enhanced at the cathode-facing pole of the cell (depolarization) and suppressed at the anode-facing pole (hyperpolarization).

**Figure 5:**
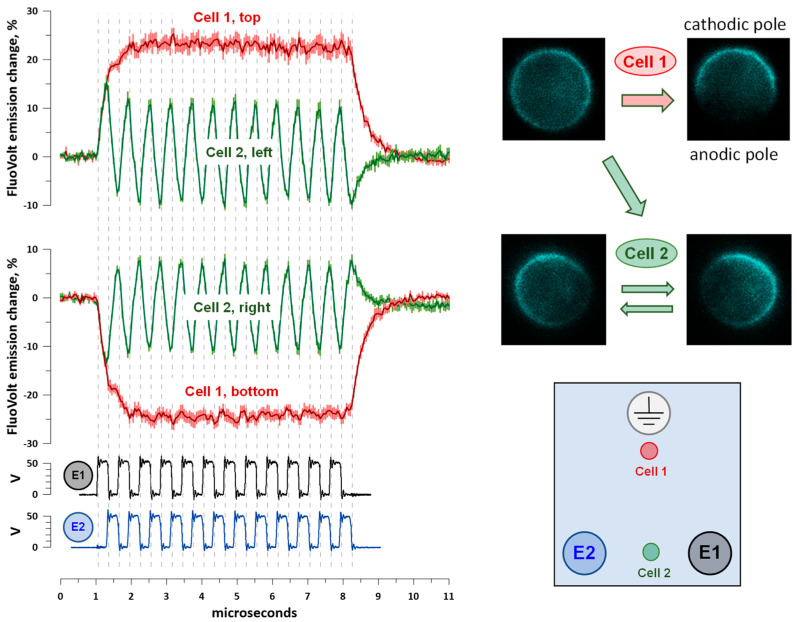
Cell membrane charging by 300 ns pulses applied alternately to base electrodes E1 and E2 of a triangular electrode array (inset). Pulses were delivered at 300 ns intervals (at 1.67 MHz). Oscilloscope traces of pulses are shown in the bottom plots. The inactive electrode became ground, and the apex electrode was grounded all the time. The electric field at both cell locations, shown in the inset, was about 0.15 kV/cm. In FluoVolt-loaded CHO cells, pulses enhanced fluorescence at the cathode-facing side and suppressed it at the anode-facing side (inset). Plots show changes in FluoVolt emission measured at the opposite sides of cells in the direction of the maximum electric field gradient (top and bottom sides for Cell 1 location; left and right sides for Cell 2 location). Each plot is built from 220 measurements taken at 50 ns intervals and averaged across 5 cells (mean +/− s.e.). See text for more details.

Cells placed either near the apex electrode or between base electrodes of a triangular array (Section 4.2) were stimulated by 300 ns pulses applied in alternation to the base electrodes. The apex electrode and the non-energized base electrode served as grounds. A total of 12 pulses were applied to each of the base electrodes, with 300 ns inter-pulse intervals (Figure 5). The pulse amplitude was set to 50 V so that the maximum electric field at the cell location (~0.15 kV/cm) remained below the electroporation threshold and multiple stimulation cycles produced the same membrane response (see Section 4.5 for details.)

Cells placed between the base electrodes displayed sawtooth-like changes in membrane potential in synchrony with the applied pulses (Figure 5). Depolarization at the cathode-facing pole of the cell manifested as a ~10% emission increase, and hyperpolarization at the anode-facing pole caused a ~10% emission decrease. With the charging time constant of about 200 ns in an average-sized CHO cell [17], 300 ns pulses charged the membrane only partially, followed by a forced discharge when the electric field direction changed and a new partial charging afterwards.

In stark contrast, the cells near the apex reached a much higher polarization (~25% change in FluoVolt emission), which followed the envelope of the pulse train but not the individual pulses of it. More than a twofold larger polarization that also lasts as long as the entire train will, of course, be far more efficient at electroporation and electrostimulation than small and brief alternating de- and hyperpolarizations near the base electrodes. One can expect that reducing pulse duration further will reduce polarizations at the base electrodes even more, without changing polarization at the apex electrode (because individual pulses coalesce at the apex). Reducing pulse duration and increasing train duration should enable the most efficient electrostimulation or electroporation at the apex while avoiding any effects at the base electrodes.

## 3. Discussion

Our study quantified how the efficiency of electroporation with nsEPs depends on the change in the electric field direction. To our knowledge, previous studies were limited to 90° angle change only and were performed with “long” conventional pulses at low repetition rates. These studies did not explore bipolar cancellation effects and focused primarily on the enhancement of electroporation or transfection when the cell membrane was electroporated from orthogonal directions.

Indeed, it makes sense that electroporating a larger area of the membrane can produce more pores and enhance electroporative transport across the membrane. Moreover, applying pulses to an already electroporated region of the membrane may charge it less efficiently than the intact membrane, which would gradually decrease the electroporation efficiency when multiple co-directional pulses are applied. However, experiments with nsEP, at least at “mildly electroporating” electric field strengths, did not confirm these expectations. Cross-directional pulses (applied at 90° angle change) and pulses applied at smaller angles were just as efficient as co-directional pulses (Figure 2), no matter that the cross-directional pulses electroporated a larger portion of the membrane and produced less damage per unit of the affected membrane area (i.e., the membrane remained “more intact” and should be more susceptible to electroporation). This result, although unexpected, is consistent with prior findings that electroporative dye uptake increases in a linear proportion to the number of nsEPs applied, as if each of them independently opens a certain number of pores [29]. The linear dependence on pulse number was also noted in some [30] but not other [31] studies with “long” electroporation pulses. Additionally, rotating the electric field in CANCAN remote stimulation did not enhance electroporation in the center of a quadrupole array, even though pulses were delivered from four orthogonal directions (Figure 5 in Pakhomov et al. [24]). Apparently, an increase in membrane conductance due to a “mild” electroporation is not sufficient to impede membrane charging, even if pulses come from the same direction as the previous pulses to an already electroporated membrane area. This hypothesis appears consistent with our research in progress (unpublished) with strobe imaging of the membrane potential at the electric field strengths above the electroporation threshold.

The strong dependence of electroporation on the angle between nsEP vectors has led to a unique “unipolar” pattern of electroporation in triangular electrode arrays. Electroporation was restricted to the area near the apex electrode, which was never energized and served as ground for the pulses applied to the base electrodes. The electroporated cells formed a comet-shaped region pointing from the apex electrode towards the base. The diverse medical applications of electroporation may benefit from achieving an effect locally at the target while avoiding any adverse effects, such as electroporation and neuromuscular stimulation, at the return electrode(s) placed away from the target. This is usually achieved by increasing the size of the return electrode(s) and thereby reducing the local current density to values near or below threshold. Using a triangular electrode array offers a novel and efficient method of restricting the effects to just one electrode placed at the target and eliminating them at the other two electrodes (which can still be made large in size to reduce the current density and take advantage of both approaches).

**Figure 6:**
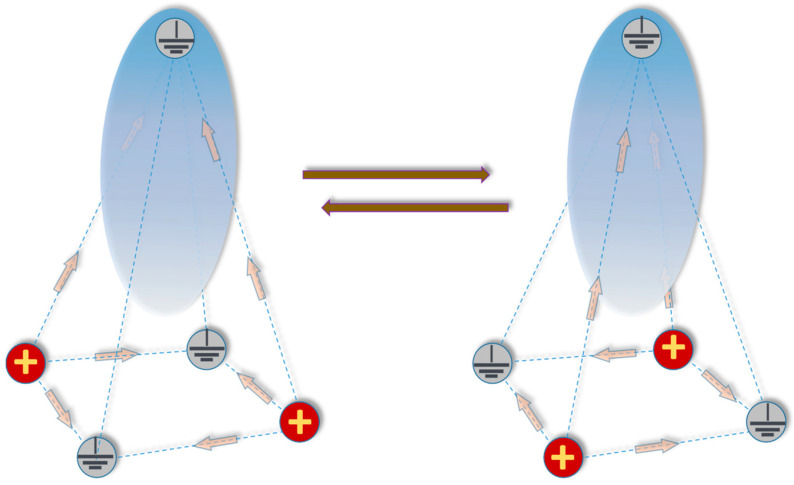
A schematic explaining the pyramid electrode configuration for selective focal electrostimulation or electroporation. Diagonal pairs of base electrodes are energized in alternation, while the pair not energized serves as ground. The apex electrode serves as ground all the time. Arrows show main directions of the electric field. Note that the arrows in the left and right panels are counter-directional at the base but co-directional towards the apex. A semi-transparent blue ellipsoid approximates the volume where effects are expected, strongest near the apex electrode and gradually weakening towards the base. See text for more details.

In a 3D environment, a triangular electrode array can be upgraded into a pyramid electrode configuration (Figure 6) where the apex electrode is ground while diagonal pairs of base electrodes are energized in turns by unipolar nsEPs. Identically to the triangular array, individual nsEPs applied to the diagonal pairs of base electrodes become co-directional and coalesce into a “long” pulse near the apex, but remain counter-directional and inefficient at the base. The pyramid configuration likely provides even better suppression of effects at the base than the triangle one, pending confirmation by direct experiments.

Electroporation and electrostimulation treatments of deep-seated tissues and tumors may involve the placement of one electrode at the target while the return electrode(s) stay on the skin. In this case, triangle and pyramid configurations offer an additional benefit of better penetration through skin, the most resistive barrier. Human skin resistance drops nearly 1000-fold when the frequency is increased from 100 Hz to 1 MHz [32], making it a high-pass filter. Nanosecond pulses, in particular bipolar nsEPs at high repetition rates, contain a larger proportion of high-frequency harmonics [33], thus resulting in reduced losses in skin. Overall, we expect that new electrode configurations and protocols, especially the pyramid configuration with short nsEPs applied to base electrodes, will find applications for selective electroporation and electrostimulation at the target while avoiding unwanted side effects elsewhere.

## 4. Materials and Methods

### 4.1. Cell Culture

The experiments in cell monolayers (Section 2.1, Section 2.2 and Section 2.3) were performed in bovine pulmonary artery endothelial (BPAE) cells, a gift from Dr. J. Catravas (Center for Bioelectrics, ODU). The studies of cell membrane charging using strobe photography (Section 2.4) were performed in CHO-K1 cells purchased from the American Type Culture Collection (ATCC, Manassas, VA, USA). Same as in the previous studies [17,34], CHO cells were selected because they do not express endogenous voltage-gated channels and retain a round shape for several hours after seeding on a coverslip.

The culture conditions and protocols for both cell lines were described recently [17]. In brief, the BPAE cells were cultured in a low-glucose DMEM medium with 2.5 μg/mL amphotericin B (Thermo Fisher Scientific, Waltham, MA, USA), 100 I.U./mL penicillin, 0.1 mg/mL streptomycin (Gibco, Gaithersburg, MD, USA), and 10% fetal bovine serum (Atlanta Biologicals, Norcross, GA, USA) at 37 °C with 5% CO_2_ in air. The cells were harvested 12–18 h before the experiments and then transferred at 2 mL/dish, (0.3–0.6) × 10^6^ cells/mL, into 35 mm glass-bottomed culture dishes (MatTek, Ashland, MA, USA) pre-coated with 0.2% gelatin. The CHO cells were propagated in Ham’s F12K medium (Mediatech Cellgro, Herdon, VA, USA) with the same supplements as for BPAE cells, except for amphotericin B. The cells were transferred into 35 mm glass-bottom culture dishes 1–2 h before measurement.

### 4.2. Electric Pulse Treatments

Brief trains of unipolar, nearly rectangular 300 or 600 ns EPs were delivered from an EPULSUS-FPM4-7 pulse generator [35]. To produce trains of paired pulses, a second channel of the generator was programmed to generate an identical pulse, either with no delay or with a brief 50 ns delay after the pulse from the first channel. For the experiments described in Section 2.1, Section 2.3 and Section 2.4, these two channels were connected to two base electrodes in a triangular electrode array. Whenever the 2nd channel was energized, the 1st channel became ground, and vice versa; the apex electrode was connected to ground all the time. Energizing the base electrodes in alternation produced the electric field whose vector direction changed with different angles depending on the location within the array (Figure 1). Counter-directional pulses at the base of the array (180° angle change) gradually turned into co-directional pulses towards the apex. Applying trains of paired pulses resulted in swinging of the electric field vector direction.

**Figure 7:**
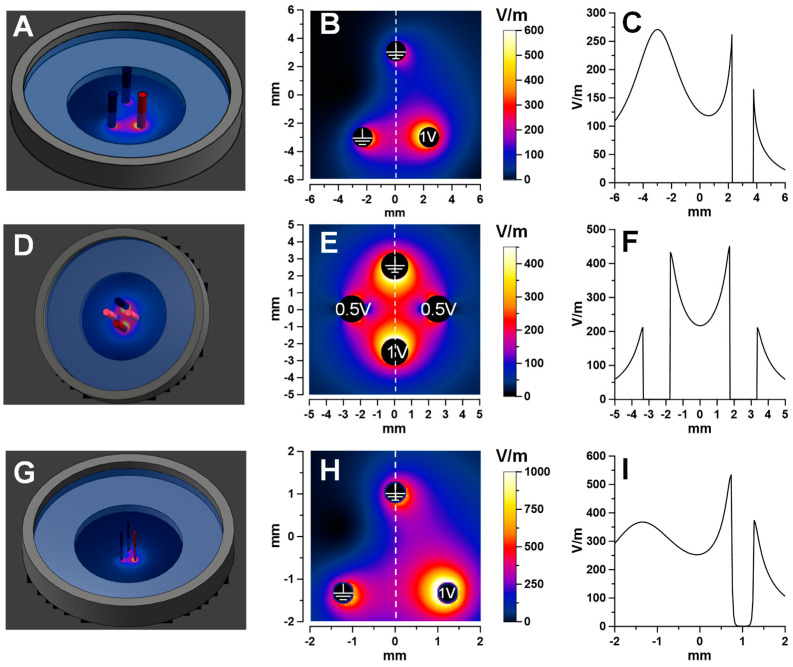
Electric field simulation for the three electrode configurations employed in the study. Panels (**A**,**D**,**G**) illustrate the models of glass-bottom Petri dishes with electrodes placed orthogonally to the bottom. Panels (**B**,**E**,**H**) show the respective electric field distribution maps in the plane 1 µm above the bottom of the dish filled with a 1.6 S/m solution when 1 V was applied to one of the electrodes (1 V and 0.5 V in (**E**)). Panels (**C**,**F**,**I**) show the electric field strength profiles along the dashed lines in the respective panels on the left.

The triangular electrode array for nsEP treatments of cell monolayers (Section 2.1 and Section 2.3, Figure 7A–C) was made of three blunt hollow stainless-steel needles with outside and inside diameters of 1.5 and 0.5 mm (Integrated Dispensing Solutions, Agoura Hills, CA, USA). They were placed orthogonally to the monolayer and lowered using an MX130L micromanipulator (Siskiyou Corporation, Grants Pass, OR, USA) until the tips touched the glass bottom of the culture dish.

Cell monolayer experiments focused on the 90° vector change only (Section 2.2) utilized a 4-electrode square array (Figure 7D–F). To electroporate cells in the center of the array, one electrode was energized from channel 1 of the generator, and the diagonally opposite electrode was connected to ground (Figure 7E). Two other electrodes, at the sides of the array in Figure 7E, were simultaneously energized from the 2nd channel to 50% of the voltage, with the sole purpose to make the electric field in areas near the center of the array more uniform (cell fluorescence was measured in a 200 µm square ROI in the center, see below). Applying all the same settings with a 90° turn produced a cross-directional electric pulse. The array was made of 1.5 mm hollow needles and positioned in touch with the monolayer.

The membrane charging experiments (Section 2.4, Figure 7G–I) utilized a triangular array with electrodes made of 0.5 mm tungsten rods (A-M Systems, Sequim, WA, USA). It was positioned orthogonally to the bottom of the culture dish, with the electrode tips 50 µm above it.

The pulse shapes and amplitudes were continuously monitored with a TDS 3052 oscilloscope (Tektronix, Beaverton, OR, USA). Sample traces of the pulses are shown in insets in Figure 4.

The maximum local heating from the nsEP treatments was estimated using the adiabatic heat equation [36]. For the maximum number of pulses and at the strongest electric field area, heating did not exceed 4.3 °C.

### 4.3. Numerical Simulation of the Electric Field Distribution

Numerical simulations of the electric field distribution were performed with a finite element solver Sim4Life V5.2 (Zurich Med Tech, Zurich, Switzerland), as reported previously [17,23,37]. The electrodes were modeled as parallel cylinders perpendicular to the glass bottom of a 35 mm diameter plastic dish (Figure 7). The dish was filled with a 1.6 S/m solution to a depth of 3.5 mm. The model was meshed with approximately 18 million cells, with a 30 µm minimum voxel size. The electric field values were calculated in the plane 1 µm above the glass, using a Low-Frequency Electro Ohmic Quasi-Static solver for 1 V or 0.5 V applied to the active electrodes (Figure 7B,E,H). ROIs for measuring fluorescence were placed along the dashed lines; the electric field values along these lines are plotted separately in Figure 7C,F,I.

### 4.4. Protocols and Quantitation of Electroporation in Cell Monolayers

The protocols for cell staining and data analysis were the same as described in detail recently [17]. The BPAE cell monolayers were placed in a physiological solution (PS) composed of (in mM): 140 NaCl, 5.4 KCl, 2 CaCl_2_, 1.5 MgCl_2_, 10 D-glucose, and 10 HEPES (pH 7.4, 290–300 mOsm/kg, 1.6 S/m). All chemicals were purchased from Sigma-Aldrich (St. Louis, MO, USA). Dead cells present in the monolayer were labeled by a 10 min incubation in the PS containing 5 µg/mL of propidium iodide; this prevented the subsequent labeling of these cells by the membrane integrity marker dye YO-PRO-1 (YP, from Thermo Fisher Scientific, Waltham, MA, USA). The cells were counterstained with MemBrite Fix 405/430 (Biotium Inc., Fremont, CA, USA), which labels all cells by binding to cell surface proteins. The cells were placed in the PS with 1 µM YP 1–2 min prior to electroporation and incubated in it for 10 min after the electroporation.

The monolayer was imaged with an IX83 microscope (Olympus America, Hamden, CT, USA) equipped with an automated MS-2000 scanning stage (ASI, Eugene, OR, USA), an X-Cite 110LED illuminator (Excelitas Technologies Corporation, Waltham, MA, USA), and an ORCA-Flash4 sCMOS camera (Hamamatsu, Shizuoka, Japan). CellSens software (version 3.2, Olympus America) was utilized for automated image acquisition, filter cube selection, stage repositioning, and synchronization with illumination and camera operation. The final high-resolution image of the entire sample was generated by stitching together 216 images captured with DAPI (MemBrite) and FITC (YP) filter cubes using a 10×, 0.38NA objective.

The fluorescence intensity of the dyes was measured with MetaMorph 7.8.13 (Molecular Devices, Foster City, CA, USA). For the experiments described in Section 2.1, a total of 32 ROIs (200 × 200 µm) were placed along the dashed line (Figure 7B). The electric field non-uniformity within any ROI was <20%. In experiments with the square electrode array (Section 2.2, Figure 6E), we used data from a single 200 × 200 µm ROI placed in the center of the array. To account for “spontaneous” YP uptake, measurements were also performed within the hollow electrodes’ footprints, where cells were shielded from the electric field. Additional details of the nsEP exposure protocol and data analysis are provided in the respective Results sections and figure captions.

Grapher 16 (Golden Software, Golden, CO, USA) was utilized for data plotting and fitting. A 3D rendering of YP uptake distribution (Figure 4C) was prepared with SlideBook 6.0.12 (3i, Denver, CO, USA).

### 4.5. Strobe Imaging of Cell Membrane Charging by Electric Pulses

The method of strobe photography, aimed at resolving membrane charging and relaxation kinetics with nanosecond resolution, was introduced and validated recently [34]. In the present study, strobe photography was performed using the same equipment and protocols as described in our previous report [17]. In short, CHO cells were loaded with a FluoVolt dye (Thermo Fisher Scientific) and illuminated by ~6 ns, 440 nm laser flashes, which were delivered in synchrony with nsEPs. The time interval between a laser flash and nsEP was decreased or increased in 50 ns steps, and one photo of the stimulated cell was collected at each time interval. The 11 µs long plots in Figure 5 were built from 220 individual photos. The nsEP amplitude was set at a level below the electroporation threshold to avoid membrane damage and ensure a similar response to multiple stimuli. Dye bleaching and the variability of laser power were compensated by averaging the data collected with time interval increments and decrements and by normalizing the emission changes at the cell poles to the whole-cell emission [17].

## Data Availability

The data presented in this study are available on request from the corresponding author.
